# Supplementary material for: Strategies to improve patient loyalty and medication adherence in Syrian healthcare setting: The mediating role of patient satisfaction
Source: PLoS One. 2022 Nov 18;17(11):e0272057. doi: 10.1371/journal.pone.0272057 (PMC9674161; doi:10.1371/journal.pone.0272057)
Supplement: S1 File — (PDF) [file pone.0272057.s001.pdf]

Normality Test of Constructs

Construct	Skewness	Kurtosis
Tangibility	-1.041	1.360
Empathy	-1.075	1.554
Assurance	-0.427	0.138
Reliability	0.330	-0.337
Responsiveness	0.068	-0.147
Financial Aspect	-0.657	0.022
Patient Satisfaction	-0.412	-0.132
Patient Loyalty	0.015	-0.379
Medication Compliance	-0.274	-0.461

Descriptive Statistics of Service Quality Dimensions

Construct	Dimension	Item	Mean	SD
Service quality	Tangibility	T1	3.782	0.993
		T2	3.885	0.99
		T3	3.717	1.028
		T4	3.62	1.041
		T5	3.844	0.957
		T6	3.698	0.927
	Empathy	E1	3.791	1.031
		E2	3.548	0.237
		E3	3.479	0.243
		E4	3.834	0.235
		E5	3.882	1.004
		E6	3.735	0.955
		E7	3.826	0.996
	Assurance	ASSU1	3.006	1.076
		ASSU2	3.511	0.961
		ASSU3	3.47	0.835
		ASSU4	3.548	0.867
		ASSU5	3.411	0.995
	Reliability	REL1	2.517	1.053
		REl2	2.445	1.113
		REl3	2.797	0.743
		REL4	2.461	1.065
		REL5	2.439	1.055
		REL6	2.53	1.047
		REL7	2.405	0.991
	Responsiveness	RES1	3.202	0.947
		RES2	3.199	0.919
		RES3	3.256	0.764
		RES4	3.072	0.909
	Financial Aspect	FA1	3.193	1.008
		FA2	3.212	1.061
		FA3	3.271	0.998
		FA4	3.097	1.041
		FA5	3.445	0.988

Descriptive Analysis of Patient Satisfaction, Patient Loyalty, and Adherence

Construct	Item	Mean	SD
Patient Satisfaction	PS1	3.271	1.037
	PS2	3.019	1.017
Patient Loyalty	PL1	3.411	0.97
	PL2	3.477	0.937
Medication Compliance	MC1	3.128	1.113
	MC2	3.224	1.07
	MC3	3.417	0.967
	MC4	3.464	0.95

Discriminant Validity Using Cross Loading-Values

	ASSU	E	FA	MA	PL	PS	RL	RES	T
ASSU1	0.764	0.333	0.402	0.353	0.39	0.348	0.361	0.377	0.335
ASSU2	0.876	0.534	0.421	0.342	0.439	0.397	0.347	0.323	0.532
ASSU3	0.858	0.45	0.398	0.298	0.326	0.354	0.203	0.228	0.402
ASSU4	0.833	0.356	0.364	0.344	0.327	0.409	0.235	0.211	0.357
ASSU5	0.86	0.428	0.425	0.308	0.322	0.433	0.335	0.205	0.425
E1	0.45	0.85	0.313	0.416	0.366	0.296	0.304	0.341	0.74
E2	0.389	0.847	0.358	0.42	0.345	0.329	0.297	0.231	0.778
E3	0.383	0.751	0.209	0.3	0.279	0.274	0.272	0.207	0.668
E4	0.409	0.787	0.334	0.37	0.28	0.305	0.216	0.185	0.52
FA1	0.447	0.332	0.892	0.425	0.374	0.444	0.289	0.254	0.297
FA2	0.442	0.355	0.875	0.453	0.345	0.423	0.282	0.252	0.33
FA3	0.31	0.191	0.845	0.401	0.278	0.446	0.408	0.269	0.194
FA4	0.294	0.2	0.765	0.431	0.289	0.32	0.287	0.172	0.15
FA5	0.465	0.462	0.73	0.406	0.373	0.411	0.323	0.226	0.447
MA1	0.193	0.298	0.431	0.816	0.409	0.28	0.445	0.389	0.244
MA2	0.224	0.318	0.352	0.819	0.291	0.312	0.397	0.3	0.254
MA3	0.444	0.514	0.446	0.83	0.42	0.45	0.342	0.356	0.522
MA4	0.4	0.386	0.447	0.815	0.461	0.453	0.401	0.376	0.431
PL1	0.389	0.385	0.344	0.403	0.888	0.505	0.334	0.489	0.413
PL2	0.391	0.329	0.383	0.471	0.911	0.394	0.5	0.616	0.388
PS1	0.47	0.369	0.477	0.4	0.425	0.893	0.279	0.237	0.345
PS2	0.361	0.297	0.416	0.426	0.463	0.896	0.39	0.23	0.369
REL1	0.278	0.228	0.398	0.406	0.389	0.34	0.832	0.329	0.273
REL2	0.189	0.228	0.328	0.369	0.331	0.321	0.835	0.296	0.271
REL3	0.365	0.326	0.318	0.432	0.391	0.311	0.836	0.277	0.346
REL4	0.32	0.33	0.309	0.472	0.47	0.318	0.877	0.37	0.335
REL5	0.361	0.276	0.333	0.381	0.397	0.331	0.854	0.327	0.3
REL6	0.286	0.317	0.27	0.371	0.385	0.279	0.845	0.297	0.286
RES1	0.348	0.305	0.275	0.393	0.498	0.304	0.352	0.893	0.324
RES2	0.282	0.266	0.252	0.358	0.545	0.189	0.302	0.904	0.268
RES3	0.219	0.219	0.226	0.39	0.575	0.192	0.329	0.826	0.294
T1	0.419	0.688	0.32	0.404	0.412	0.357	0.346	0.318	0.847
T2	0.407	0.818	0.356	0.436	0.373	0.369	0.303	0.278	0.874
T3	0.399	0.715	0.322	0.384	0.365	0.323	0.307	0.252	0.888
T4	0.441	0.729	0.325	0.387	0.386	0.336	0.354	0.288	0.896
T5	0.331	0.505	0.134	0.26	0.274	0.302	0.196	0.256	0.703
T6	0.465	0.726	0.265	0.382	0.405	0.32	0.273	0.307	0.806

R-Square and R-Square Adjusted Values

Construct	R Square	R Square Adjusted
Medication adherence	0.462	0.45
Patient loyalty	0.544	0.533
Patient satisfaction	0.352	0.34

Result of PLS-Predict

Item	PLS RMSE	LM RMSE	PLS-LM	Q^2_ predict
MA4	0.791	0.832	-0.041	0.310
MA3	0.795	0.806	-0.011	0.329
MA2	0.959	0.968	-0.009	0.202
MA1	0.948	0.909	0.039	0.279
PL2	0.747	0.740	0.007	0.463
PL1	0.865	0.890	-0.025	0.309
PS1	0.800	0.811	-0.011	0.277
PS2	0.848	0.882	-0.034	0.239
