# Supplementary material for: Strategies to improve patient loyalty and medication adherence in Syrian healthcare setting: The mediating role of patient satisfaction
Source: PLoS One. 2022 Nov 18;17(11):e0272057. doi: 10.1371/journal.pone.0272057 (PMC9674161; doi:10.1371/journal.pone.0272057)
Supplement: Attachment — Submitted filename: Response to Reviewers-submit.pdf [file pone.0272057.s003.pdf]

Dear Editor-in-Chief,

Thank you for giving the opportunity to revise our manuscript entitled “Strategies to improve patient loyalty and medication adherence in Syrian healthcare setting: The mediating role of patient satisfaction”. The manuscript had been revised based on the suggestions given by the Reviewers.

We appreciate the interest that Editor has taken in the submitted manuscript and the constructive comments He has given. However, we will do our utmost to answer any other questions from the Reviewers.

The authors express profound gratitude for providing a full publication fee waiver offered by PLOS.

Regards

Authors

==

Answers- Reviewer A- Comment:

Author would like to take this opportunity to thank Reviewer for the effort and expertise to review manuscript. Author had rewritten the manuscript with accuracy, brevity and clarity. The manuscript had been organized properly, keeping in mind fully incorporate reviewer’s suggestions into a revised manuscript and respect the requirements of the journal.

Answers- Reviewer A- Comment:

The research is well-designed and served purpose of the study. The aim of study was to examine the relationships between service quality, patient satisfaction, patient loyalty and medication adherence in the Syrian healthcare system.

The work is well-written however; some minor changes are required before acceptance:

- In literature review, compare your work with reported work, highlighting main advantages of your work.

  Recent previous literatures were added as a foundation and as support for a new insight that we contribute for (2.1 relationship between SQ, PS and PL) and for (2.2 relationship between SQ, PS and MA). The literature reviews also provide a solid background for our research paper’s investigation.

We also highlighted the significance of our research work by clarifying and explaining the main gaps within a specific subject area. For example, the modification of SERVQUAL model, the reason behind including the financial aspect component (we summarize and synthesize the arguments and ideas of the scholars). The mediating impact of PS on the relationship between SQ and MA.

- Conclusion is not comprehensive; it should be precise and targeted the major results obtained.

  The conclusion section had been improved to include all the main results (based on the findings of the proposed hypotheses).

- In research methodology, a total of 600 questionnaires were distributed, the returned were (410) questionnaires with response rate (68.33%)…….. Why 410 and 68.33% are mentioned in bracket? It makes sense in this sentence…… As depicted in Table 1, there were 106 (33.02%) female and 215 (66.97%) male patients. In all other places modify it.

  As requested, the brackets were removed from this part: A total of 600 questionnaires were distributed, the returned were 410 questionnaires with response rate 68.33%. However, 89 questionnaires were excluded because of missing data or not correctly filled. While, the brackets in this paragraph were kept (the number outside the bracket reflects the frequency, while the number inside the brackets reflects the percentile): Table 1 reflects the demographic information of the patients who participated in this study. As depicted in Table 1, there were 106 (33.02%) female and 215 (66.97%) male patients. The majority of the patients came from the age group of 41-50 years old (40.81%). Most of them were single 197 (61.37%) whereas 124 (38.63%) of them were married. In addition, most of the patients were visited the hospital for the second time 211 (65.73%) followed by those who had visited hospital for three times 93 (28.97%).

- Exclusion criteria should be better explained.

  The exclusion criteria were clarified: During data collection….; Besides, we identified very few outliers, then….; Several procedural techniques were used to minimize non-response….; Besides, we informed the patients….

- Research implications and recommendations section is too lengthy. Make it short with relevant data.

  Done.

==

Answers- Reviewer B- Comment:

Author would like to take this opportunity to thank Reviewer for the effort and expertise to review manuscript. Author had rewritten the manuscript with accuracy, brevity and clarity. The manuscript had been organized properly, keeping in mind fully incorporate reviewer's suggestions into a revised manuscript and respect the requirements of the journal.

==
