# Supplementary material for: Strategies to improve patient loyalty and medication adherence in Syrian healthcare setting: The mediating role of patient satisfaction
Source: PLoS One. 2022 Nov 18;17(11):e0272057. doi: 10.1371/journal.pone.0272057 (PMC9674161; doi:10.1371/journal.pone.0272057)
Supplement: Attachment — Submitted filename: comments 2.docx [file pone.0272057.s002.docx]

The research is well-designed and served purpose of the study. The aim of study was to examine the relationships between service quality, patient satisfaction, patient loyalty and medication adherence in the Syrian healthcare system.

The work is well-written however; some minor changes are required before acceptance:

- In literature review, compare your work with reported work, highlighting main advantages of your work.
- Conclusion is not comprehensive; it should be precise and targeted the major results obtained.
- In research methodology, a total of 600 questionnaires were distributed, the returned were (410) questionnaires with response rate (68.33%)…….. Why 410 and 68.33% are mentioned in bracket? It makes sense in this sentence…… As depicted in Table 1, there were 106 (33.02%) female and 215 (66.97%) male patients. In all other places modify it.
- Exclusion criteria should be better explained.
- Research implications and recommendations section is too lengthy. Make it short with relevant data.
